# Assessment of the Effect of Transcranial Direct Current Stimulation (tDCS) on Insomnia in NHS Patients

**DOI:** 10.1192/bjo.2026.11055

**Published:** 2026-06-30

**Authors:** Chris Griffiths, Joanne Warcaba, Sinead Galvin, Helen MacMillan, Kate Walker

**Affiliations:** 1 Northamptonshire Healthcare NHS Foundation Trust, Northampton, United Kingdom; 2 General Practice Alliance, Northampton, United Kingdom

## Abstract

**Aims:**

Transcranial direct current stimulation (tDCS) is a non-invasive brain stimulation technique used at home and delivers mild electric currents, applied through two pads on the forehead. It does not have the side effects associated with antidepressant medication, is highly acceptable and easily offered through primary care. Research studies show that tDCS can significantly improve sleep quality and provide insomnia remission. Rates of insomnia remission of between 22–50% have been seen. The aim of the project was to assess effect of ‘Flow’ tDCS on insomnia in primary care patients.

**Methods:**

Intervention: tDCS for 30 minutes, for five sessions per week for four weeks. Baseline and follow-up scores collected. Interviews with GPs and 14 participants about their experience.

**Results:**

Significant improvements on 1. Sleep: Insomnia Severity Index (ISI); 2. Depression: Patient Health Questionaire-9 (PHQ-9); 3. cognitive functioning: Perceived Deficits Questionnaire (PDQ-5); Quality of Life (EQ-5D-5L); and real-world functioning: Work and social adjustment (WASA). Patients have described improved quality of life, reduction in insomnia symptoms, improved sleep, improved mood, better meaningfulness/engagement in life, and better educational, social and work functioning. The project showed that offering tDCS was feasible and effective.

**Conclusion:**

Evidence about the improvement with tDCS could expand NHS treatment for millions of people who experience insomnia. Having a new effective treatment in the NHS that can be used at home could be life-changing for people with insomnia. Providing a non-pharmacological choice in primary care is essential.

